# Genome-Wide Identification and Expression Profiling of Germin-Like Proteins Reveal Their Role in Regulating Abiotic Stress Response in Potato

**DOI:** 10.3389/fpls.2021.831140

**Published:** 2022-02-17

**Authors:** Madiha Zaynab, Jiaofeng Peng, Yasir Sharif, Mahpara Fatima, Mohammed Albaqami, Rashid Al-Yahyai, Khalid Ali Khan, Saqer S. Alotaibi, Ibrahim A. Alaraidh, Hassan O. Shaikhaldein, Shuangfei Li

**Affiliations:** ^1^Shenzhen Key Laboratory of Marine Bioresource and Eco-Environmental Sciences, College of Life Sciences and Oceanography, Shenzhen University, Shenzhen, China; ^2^Instrument Analysis Center, Shenzhen University, Shenzhen, China; ^3^College of Plant Protection, Fujian Agriculture and Forestry University, Fuzhou, China; ^4^College of Agriculture, Fujian Agriculture and Forestry University, Fuzhou, China; ^5^Department of Biology, Faculty of Applied Science, Umm Al-Qura University, Makkah, Saudi Arabia; ^6^Department of Plant Sciences, College of Agricultural and Marine Sciences, Sultan Qaboos University, Muscat, Oman; ^7^Research Center for Advanced Materials Science (RCAMS), King Khalid University, Abha, Saudi Arabia; ^8^Unit of Bee Research and Honey Production, Faculty of Science, King Khalid University, Abha, Saudi Arabia; ^9^Department, Faculty of Science, King Khalid University, Abha, Saudi Arabia; ^10^Department of Biotechnology, College of Science, Taif University, Taif, Saudi Arabia; ^11^Botany and Microbiology Department, Science College, King Saud University, Riyadh, Saudi Arabia

**Keywords:** abiotic stress, gene expression, gene structure, miRNA, RNA-seq, stress responses

## Abstract

Germin and germin-like proteins (GLPs) perform a significant role in plants against biotic and abiotic stress. To understand the role of GLPs in potato, a comprehensive genome-wide analysis was performed in the potato genome. This study identified a total of 70 *StGLPs* genes in the potato genome, distributed among 11 chromosomes. Phylogenetic analysis exhibited that *StGLPs* were categorized into six groups with high bootstrap values. *StGLPs* gene structure and motifs analysis showed a relatively well-maintained intron–exon and motif formation within the cognate group. Additionally, several *cis*-elements in the promoter regions of *GLPs* were hormones, and stress-responsive and different families of miRNAs target *StGLPs*. Gene duplication under selection pressure also exhibited positive and purifying selections in *StGLPs*. In our results, the *StGLP5* gene showed the highest expression in response to salt stress among all expressed *StGLPs*. Totally 19 *StGLPs* genes were expressed in response to heat stress. Moreover, three genes, *StGLP30*, *StGLP17*, and *StGLP14*, exhibited a relatively higher expression level in the potato after heat treatment. In total, 22 genes expressed in response to abscisic acid (ABA) treatment indicated that ABA performed an essential role in the plant defense or tolerance mechanism to environmental stress. RNA-Seq data validated by RT-qPCR also confirm that the *StGLP5* gene showed maximum expression among selected genes under salt stress. Concisely, our results provide a platform for further functional exploration of the *StGLPs* against salt and heat stress conditions.

## Introduction

Germin-like proteins (GLPs) are pervasive proteins regarded as water-soluble glycoproteins present in either cell apoplast or its extracellular matrix (Lane et al., 1992; Dunwell et al., 2008). These proteins were first identified in wheat germinating embryos (Lu et al., 2010). At present, several proteins have been identified in different plant groups that exhibit 30–70% sequence homogeneity with wheat germin proteins (Finn et al., 2007).

Germin-like genes are expressed in embryos, leaves, stems, roots, flowers, and seeds of plants responding to various environmental stimuli based upon the species or genes under consideration (Wang et al., 2013). Abiotic and biotic stresses significantly affect plant growth and production worldwide (Raza et al., 2020; Qasim et al., 2021, 2018a; Raza, 2021). Various evidence has been given as an indication of *GLPs* involvement in defense and tolerance mechanisms of plants (Breen and Bellgard, 2010). Incidents like pathogen infections, insect infestation, or exposure to various chemicals such as salicylic acid, hydrogen peroxide, or ethylene may trigger the expression level of *GLPs* (Godfrey et al., 2007). Many internal elements have influenced the expression of germin, as in wheat, where the germin gene gf-2.8 is induced by hormone auxin (Schweizer et al., 1999). Transient overexpression and silencing of some *Hordeum vulgare GLP* genes have imparted enhanced resistance against fungal infection of powdery mildew (Schweizer et al., 1999). The promoter alternative of rice oxalate oxidase genes envisaged in the resistance against *Magnaporthe oryzae*. For some subfamilies, transitory and stable expression depicted the superoxide dismutase activity of the encoded protein (EC1.15.1.1) (Zimmermann et al., 2006). The silencing of GLPs in *Nicotiana attenuate* has proved fruitful in enhancing performance against herbivores (Lou and Baldwin, 2006). Fluctuation in the transcription of germin genes in *Brassica napus* and nearly associated *Arabidopsis* is found during the circadian cycle (Heintzen et al., 1994). These *GLPs* are associated with the protective mechanisms of cereals against the infection of fungal pathogens (Lane, 2000). Studies have revealed that the transfer of *GLPs* from soybean and sunflower to *Nicotiana tabacum* can increase its resistance against *Sclerotinia sclerotiorum* (Beracochea et al., 2015; Zhang et al., 2018). Transient-enhanced expression of *TaGLP4* and *HvGLP4* makes wheat and barley plants more resistant to *Blumeria graminis.* At the same time, the ephemeral gene that is silenced by utilizing RNA interference minimizes the basal resistance in cereals (Christensen et al., 2004, p. 4). A study is conducted to explore that the regulatory factors in various GLP gene promoters and several regulatory factors are associated with many essential roles with variable copy numbers and dispersed in locations of various positive or negative strands. Cotton GLP as *GhABP19* is involved in various essential tasks in the exhibition of resistance to *Verticillium* and *Fusarium wilt* in crops (Pei et al., 2019, p. 19). A few GLP subfamily members are intricated in a vast range of fungal disease resistance in the tissues of leaves (Davidson et al., 2009).

*Germin-like protein* genes have variable expression and multiple tasks in several tissues and at different phases of plant growth processes (Barman and Banerjee, 2015). This gene family is one of those gene families that are crucial and indispensable for the defense and stress tolerance mechanisms of plants. Expression of these genes varies against various abiotic stress such as salt (Wang et al., 2013), heavy metal (Berna and Bernier, 1999), and drought stress. Moreover, some purposes of *GLP* genes have been characterized in *Hordeum vulgare* (Zimmermann et al., 2006), *Arabidopsis thaliana* (Rietz et al., 2012), *Oryza sativa* (Li et al., 2016), and *Glycine max* (Wang et al., 2014). The expression levels of many rice *GLP* genes were altered under salt stress (Das et al., 2019). *OsGLP8-14* was found to be involved in salt stress response (Banerjee et al., 2017). Liao et al. (2021) reported the differential expression of *CsGLP* genes in response to salt, drought, and ABA treatments (Liao et al., 2021).

Potato (*Solanum tuberosum* L.) is an economically essential field crop and is widely grown as a basic food commodity worldwide. About 368,168,914 tons of potatoes were harvested in 2018 on 17,578,672 ha of land, and over 1 billion people consumed them (Rashid et al., 2021). Like other plant species, potato yield is also in jeopardy due to many biotic and abiotic environmental fluctuations (Zaynab et al., 2021d). To date, the expression analysis of *GLP* genes against abiotic stress has not been reported in potatoes. Therefore, this study used genome-wide analysis to find *GLP* genes in the potato genome. Furthermore, their evolutionary relationships, synteny analysis, gene structures, conserved motifs, *cis*-elements, and miRNA predictions were investigated. Expression profiling in many tissues/organs and response to various hormones and abiotic stresses have been substantially explored, enabling us to explore and understand these *GLPs* in extensive detail in the potato genome.

## Materials and Methods

### Identification and Characterization of GLP Genes in Potato

In this study, we used two methods to identify *GLP* genes from the genome of the *Solanum tuberosum.* The first method was the Hidden Markov Model (HMM), and the second one was the BLASTP search (Raza et al., 2021; Zaynab et al., 2021d). Potato genome sequences were retrieved from JGI Phytozome 12.0^1^ database (Goodstein et al., 2012). GLP amino acids sequences of *A. thaliana* were taken from TAIR^2^ (Rhee, 2003) database and used as a query to run BLASTP search. The HMM file with Pfam ID PF00190 containing *GLP* genes was downloaded from Pfam^3^ (El-Gebali et al., 2019) database. Moreover, a local HMMER 3.1^4^ webserver was utilized to identify the *GLP* genes with default parameters (Finn et al., 2015). Eventually, a total of 70 *GLP* genes were identified from the genome of the potato. Non-redundant genes with conserved domains were serially named through *StGLP1–StGLP70* and used for further analysis.

Physiochemical characteristics, including molecular weight and isoelectric points, were identified through ProtParam^5^ (Gasteiger et al., 2005).

### Phylogenetic, Gene Structure, Motif, and Synteny Analysis

In order to investigate the evolutionary history of the *StGLP* family genes, a phylogenetic tree was constructed among the amino acid sequences of *Solanum lycopersicum, S. tuberosum*, and *A. thaliana.* We used MEGA 7^6^ (Kumar et al., 2018) for the construction of phylogenetic tree. The neighbor-joining method was applied to make a phylogenetic tree with 1,000 bootstrap values. TBtools software^7^ was deployed to construct the gene structure of *StGLPs* (Chen et al., 2020). Conserved motifs present in *StGLPs* sequences were recognized through MEME^8^ (Bailey et al., 2009) server. For comparative synteny analysis, Circoletto Tool^9^ was used.

### Chromosomal Locations, Selection Pressure, and *Cis*-Elements Analysis

Data related to the chromosomal location was taken from general feature format (GFF) files of *S. tuberosum* genome database (PGSC Ref seq v4.03) (The Potato Genome Sequencing Consortium, 2011). The localization of 70 identified genes on chromosomes was performed by utilizing TBtools (Chen et al., 2020). To explore the putative *cis*-elements in the promoter region of *StGLPs*, we extracted a sequence of 2 Kb upstream of start codons in the genome of *S. tuberosum*. Afterward, to analyze the promoter region of each gene, we deployed them to PlantCARE^10^ (Lescot, 2002) and figures made by TBtools (Chen et al., 2020). Besides, the synonymous and non-synonymous substitution rate was computed by employing KaKs Calculator 2.0 software using the MYN procedure. Ks and Ka refer to the number of synonymous and non-synonymous substitutions concerning the synonymous non-synonymous site (Wang et al., 2010). Divergence time (*t* = Ks/2*r*) was estimated through exchange rate as (*r* = 2.6 × 10^–9^) (Li et al., 2019).

### Prediction of miRNA and Protein–Protein Interactions

For identifying miRNA targeting *StGLPs*, psRNAtarget^11^ was used with default parameters (Dai et al., 2018). For illustrating the protein--protein interaction network, the STRING 11.0^12^ server was employed, and for reference, the genome of *S. tuberosum* was used. The exhibit specifications were propped as the confidence value is 0.9 for network; and 10 for edges evidence and maximum number of interaction.

### Expression Profiling of StGLP Genes in Different Tissues

In order to investigate the expression of *StGLP* genes in various tissues and under various stress conditions, the transcriptome data were obtained from the available public database BioProject ID: Project: PRJEB2430. For the expression analysis of *StGLP* genes in different tissues and under different stresses, fragments per kilobase million (FPKM) were observed (Zaynab et al., 2021d). The obtained data were arranged according to their expression in different tissues. The observed FPKM values were utilized to construct a heatmap through TBtools after the normalization of log2 values.

### Potato Growth Conditions and Stress Treatment

The rooster variety of *S. tuberosum* was selected as the sample for these experiments, which were conducted at the experimental station of the National Institute for Genomics and Advanced Biotechnology (NIGAB), Islamabad, Pakistan. After 6 weeks of growth, the sample was treated with 150 mM NaCl for 0 and 24 h, respectively, for salt treatment. The first time point (0 h) served as a control. After NaCl treatment, leaves were taken, and samples were immediately frozen in liquid nitrogen and stored at −80°C for total RNA extraction.

### RNA Extraction and RT-qPCR Analysis

Total RNA was extracted using TransZol Up Plus RNA Kit (TransGen Biotech, Beijing, China). For cDNA synthesis, cDNA Synthesis SuperMix was purchased from the above-mentioned Chinese company. Instructions given by manufacturers were followed during the whole process. The comprehensive knowledge about reactions of RT-qPCR has been reported in our earlier published work (Zaynab et al., 2021a). In our study, the expression data were evaluated by using the 2^–ΔΔCT^ methods (Zaynab et al., 2021c), and the elongation factor 1-alpha was chosen as a housekeeping gene (Zaynab et al., 2021b). The primers used in this study are given in Supplementary Table 1.

## Results

### Identification, Phylogenetic, Gene Structure, Motif Analysis of *GLP* Genes in Potato

**FIGURE 1:**
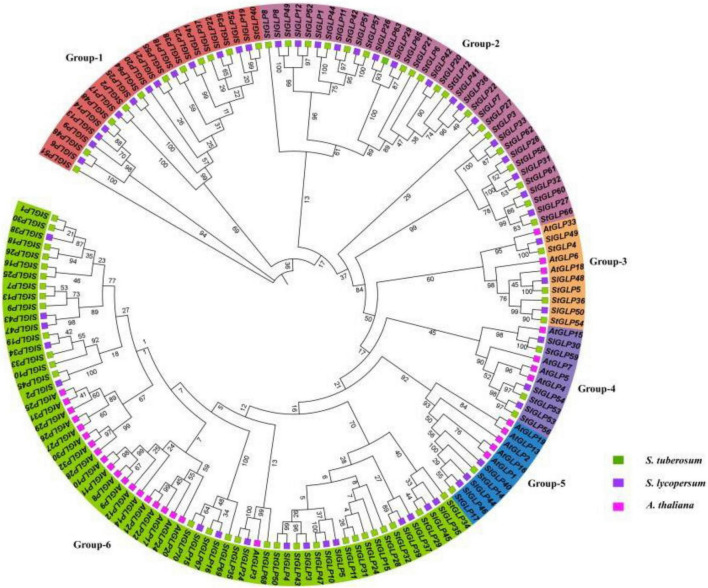
Phylogenetic tree of GLP proteins from *Arabidopsis*, *S. lycoperiscum*, and *S. tuberosum*. The colored arcs indicate different groups. The stars colors represent proteins of *Arabidopsis*, *S. lycoperiscum*, and *S. tuberosum*.

**FIGURE 2:**
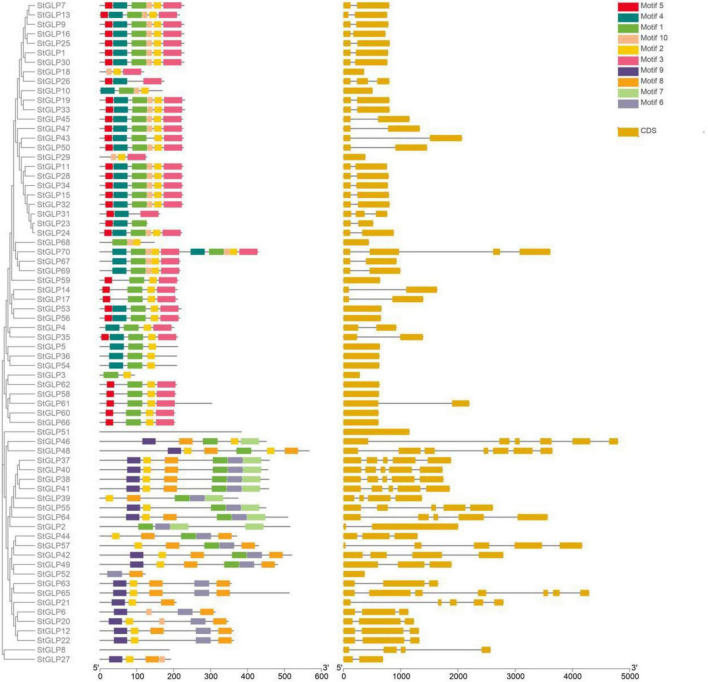
Distribution of exons, introns, and untranslated regions (UTR) in *StGLP* gene sequences and distributions of *de novo* MEME motifs in the StGLP proteins.

A total of 70 *GLP* genes were identified in the potato genome by employing sequences of *A. thaliana* as a query (Table 1). In this study, Batch-CD and SMART were utilized to confirm the domain with Pfam ID (PF00190). All these 70 genes contain a domain with Pfam ID (PF00190). The protein sequences of *A. thaliana*, *S. lycopersicum*, and identified StGLPs are given in Supplementary Table 2. Amino acids length varied from 94 to 567, the molecular weight from 10.650 to 65.780 kDa, and the isoelectric point values from 4.46 to 9.74 (Table 1). A phylogenetic tree was constructed using the neighbor-joining method through MEGA7 to illustrate the phylogenetic relationship among *A. thaliana, S. lycoperiscum*, and *S. tuberosum.* This study results showed that the phylogenetic tree was divided into six groups. Group-6 is the largest group, and group-3 is the smallest among all groups (Figure 1). Exons and introns are two main determinants in the gene family evolution. The detailed analysis of the phylogenetic relationship and gene composition illustration corroborated our understanding of the structures of *StGLPs* genes (Figure 2). The results revealed that numerical attributes of exon and intron in *StGLP* genes possessed high inconsistency in numbers of exons and introns (Figure 2). In this study, the *StGLP46* gene possessed the most extended sequence. Some genes such as *StGLP41* and *StGLP38* exhibited similarities in exon numbers. Additionally, *StGLP21* and *StGLP57* genes were equal in exons numbers but differed in their sequence lengths. For determining structural diversification and functional characterization of these *StGLPs*, we analyzed full-length sequences of 70 StGLPs through MEME software to localize their conserved motifs. The motif results revealed that a total of ten motifs were identified (Figure 2). The sequences of predicted motifs are given in Supplementary Table 3. All of the *StGLPs* genes contained motif 1 except genes *StGLP StGLP18, StGLP26, StGLP29, StGLP31, StGLP51, StGLP52, StGLP63, StGLP65, StGLP21, StGLP6, StGLP20, StGLP12, StGLP22, StGLP8*, and *StGLP27*. The length of the motif was varied from 21 to 50 amino acids. The highest 50 amino acids were possessed by motif 3,7, and 10, while motif 2 and 5 contained the lowest 21 amino acids. In our results, motif 4 had 39 amino acids, motif 8 had 35 amino acids, while motifs 1 and 9 had 41 amino acids in their sequence. In short, the reliability of group organization was exceedingly encouraged by analyzing conserved motif conformation, genetic structures, and phylogenetic relationship, indicating that StGLP proteins possess enormously well-sustained amino acid members and remain inside a group. Thus, it can be inferred that proteins with identical motifs and structures may possess functional similarities.

**TABLE 1:** List of identified putative *StGLPs* and their features.

Gene name	Transcript name	Gene start (bp)	Gene end (bp)	Chromosome name	Transcript end (bp)	Transcript start (bp)	No. of amino acids	Molecular weight	PI
*StGLP1*	PGSC0003DMT400040093	20,824,518	20,825,297	ST4.03ch02	20,825,297	20,824,518	228	24,389.04	7.79
*StGLP2*	PGSC0003DMT400032968	40,814,829	40,816,831	ST4.03ch02	40,816,831	40,814,829	515	61,094.18	5.12
*StGLP3*	PGSC0003DMT400081522	43,168,850	43,169,134	ST4.03ch00	43,169,134	43,168,850	94	10,650.29	7.99
*StGLP4*	PGSC0003DMT400057096	55,379,029	55,379,951	ST4.03ch07	55,379,951	55,379,029	201	22,001.22	7.71
*StGLP5*	PGSC0003DMT400033858	38,547,986	38,548,621	ST4.03ch07	38,548,621	38,547,986	211	22,055.77	5.84
*StGLP6*	PGSC0003DMT400000051	71,669,764	71,670,896	ST4.03ch01	71,670,896	71,669,764	313	34,015.31	6.9
*StGLP7*	PGSC0003DMT400034084	79,622,868	79,623,667	ST4.03ch01	79,623,667	79,622,868	228	24,491.01	5.62
*StGLP8*	PGSC0003DMT400017373	64,470,132	64,472,699	ST4.03ch01	64,472,699	64,470,132	188	19,878.96	8.52
*StGLP9*	PGSC0003DMT400034092	79,600,750	79,614,804	ST4.03ch01	79,601,534	79,600,750	228	24,517.12	5.88
*StGLP10*	PGSC0003DMT400046879	79,534,943	79,535,624	ST4.03ch01	79,535,624	79,535,115	169	18,065.87	9.74
*StGLP11*	PGSC0003DMT400017382	64,505,199	64,505,959	ST4.03ch01	64,505,959	64,505,199	225	24,736.65	5.51
*StGLP12*	PGSC0003DMT400061076	80,764,817	80,766,134	ST4.03ch01	80,766,134	80,764,817	362	38,977.83	5.75
*StGLP13*	PGSC0003DMT400053377	79,649,888	79,650,650	ST4.03ch01	79,650,650	79,649,888	216	23,186.36	5.36
*StGLP14*	PGSC0003DMT400047065	79,150,246	79,151,880	ST4.03ch01	79,151,880	79,150,246	210	22,228.7	8.44
*StGLP15*	PGSC0003DMT400017379	64,490,847	64,491,641	ST4.03ch01	64,491,641	64,490,847	225	24,708.72	9.28
*StGLP16*	PGSC0003DMT400046995	79,507,167	79,507,900	ST4.03ch01	79,507,900	79,507,167	228	24,625.34	6.17
*StGLP17*	PGSC0003DMT400047067	79,135,984	79,137,376	ST4.03ch01	79,137,376	79,135,984	211	22,272.7	7.8
*StGLP18*	PGSC0003DMT400053424	79,647,675	79,648,034	ST4.03ch01	79,648,034	79,647,675	119	13,318.31	6.9
*StGLP19*	PGSC0003DMT400046887	79,523,462	79,524,263	ST4.03ch01	79,524,263	79,523,462	230	24,753.47	8.55
*StGLP20*	PGSC0003DMT400000042	71,626,643	71,627,873	ST4.03ch01	71,627,873	71,626,643	348	38,041.87	5.74
*StGLP21*	PGSC0003DMT400088315	80,783,956	80,786,749	ST4.03ch01	80,786,749	80,783,956	207	22,017.21	7.69
*StGLP22*	PGSC0003DMT400061073	80,803,974	80,805,300	ST4.03ch01	80,805,300	80,803,974	362	38,986.74	6.04
*StGLP23*	PGSC0003DMT400017381	64,501,551	64,502,069	ST4.03ch01	64,502,069	64,501,551	128	14,095.21	5.42
*StGLP24*	PGSC0003DMT400017372	64,463,708	64,464,591	ST4.03ch01	64,464,591	64,463,714	222	24,245.05	6.95
*StGLP25*	PGSC0003DMT400046992	79,536,560	79,537,367	ST4.03ch01	79,537,367	79,536,560	228	24,522.09	6.49
*StGLP26*	PGSC0003DMT400053421	79,661,664	79,662,463	ST4.03ch01	79,662,463	79,661,664	174	18,975.74	8.89
*StGLP27*	PGSC0003DMT400000049	71,661,596	71,662,286	ST4.03ch01	71,662,286	71,661,596	192	21,734.96	5.39
*StGLP28*	PGSC0003DMT400017366	64,436,796	64,437,584	ST4.03ch01	64,437,584	64,436,796	225	24,725.77	8.77
*StGLP29*	PGSC0003DMT400088001	64,444,053	64,444,436	ST4.03ch01	64,444,436	64,444,053	127	14,195.59	9.43
*StGLP30*	PGSC0003DMT400053423	79,654,305	79,655,071	ST4.03ch01	79,655,071	79,654,305	228	24,429.06	6.49
*StGLP31*	PGSC0003DMT400017387	64,508,106	64,508,869	ST4.03ch01	64,508,869	64,508,106	162	17,779.49	5.19
*StGLP32*	PGSC0003DMT400017363	64,424,713	64,425,516	ST4.03ch01	64,425,516	64,424,713	225	24,860.8	8.94
*StGLP33*	PGSC0003DMT400046891	79,517,314	79,518,118	ST4.03ch01	79,518,118	79,517,314	230	24,628.3	8.55
*StGLP34*	PGSC0003DMT400017367	64,450,575	64,451,350	ST4.03ch01	64,451,350	64,450,575	225	24,878.8	8.4
*StGLP35*	PGSC0003DMT400058298	6,670,741	6,672,129	ST4.03ch05	6,672,129	6,670,741	211	22,544.89	8.58
*StGLP36*	PGSC0003DMT400030317	52,610,039	52,610,665	ST4.03ch12	52,610,665	52,610,039	208	21,643.96	6.82
*StGLP37*	PGSC0003DMT400019402	55,885,543	55,887,421	ST4.03ch06	55,887,421	55,885,543	459	51,649.76	8.95
*StGLP38*	PGSC0003DMT400019389	55,877,736	55,879,480	ST4.03ch06	55,879,480	55,877,736	458	51,627.42	7.32
*StGLP39*	PGSC0003DMT400008698	55,873,841	55,875,211	ST4.03ch06	55,875,211	55,873,841	374	42,039.48	9.33
*StGLP40*	PGSC0003DMT400019388	55,889,622	55,891,351	ST4.03ch06	55,891,351	55,889,622	455	51,137.24	9.1
*StGLP41*	PGSC0003DMT400019398	55,882,134	55,883,989	ST4.03ch06	55,883,989	55,882,134	457	51,399.11	6.04
*StGLP42*	PGSC0003DMT400044214	56,455,696	56,458,487	ST4.03ch09	56,458,487	56,455,696	520	58,788.33	6.59
*StGLP43*	PGSC0003DMT400044229	56,325,068	56,327,136	ST4.03ch09	56,327,136	56,325,068	226	24,986.91	5.77
*StGLP44*	PGSC0003DMT400026705	50,134,244	50,135,537	ST4.03ch09	50,135,537	50,134,244	371	41,766.35	6.43
*StGLP45*	PGSC0003DMT400044231	56,322,818	56,323,971	ST4.03ch09	56,323,971	56,322,818	224	23,985.76	5.49
*StGLP46*	PGSC0003DMT400049823	47,996,075	48,000,869	ST4.03ch09	48,000,869	47,996,075	451	50,927.94	9.05
*StGLP47*	PGSC0003DMT400044224	56,339,851	56,341,183	ST4.03ch09	56,341,183	56,339,851	225	24,724.45	5.72
*StGLP48*	PGSC0003DMT400082074	54,002,237	54,005,886	ST4.03ch09	54,005,886	54,002,237	567	65,780.62	7.54
*StGLP49*	PGSC0003DMT400026712	50,168,038	50,169,925	ST4.03ch09	50,169,925	50,168,038	481	54,427.26	6.82
*StGLP50*	PGSC0003DMT400096380	56,330,056	56,331,514	ST4.03ch09	56,331,514	56,330,056	226	25,186.3	9.17
*StGLP51*	PGSC0003DMT400044227	56,334,086	56,338,772	ST4.03ch09	56,338,772	56,337,621	383	43,087.09	4.46
*StGLP52*	PGSC0003DMT400026650	50,132,519	50,132,890	ST4.03ch09	50,132,890	50,132,519	123	13,673.41	6.72
*StGLP53*	PGSC0003DMT400063164	53,565,261	53,565,923	ST4.03ch03	53,565,923	53,565,261	220	23,470.25	6.5
*StGLP54*	PGSC0003DMT400036428	61,605,492	61,606,118	ST4.03ch03	61,606,118	61,605,492	208	21,519.77	5.83
*StGLP55*	PGSC0003DMT400053916	7,589,586	7,592,195	ST4.03ch03	7,592,195	7,589,586	450	51,300.84	7.77
*StGLP56*	PGSC0003DMT400063166	53,567,656	53,568,309	ST4.03ch03	53,568,309	53,567,656	217	23,201.87	6.27
*StGLP57*	PGSC0003DMT400034989	639,862	644,028	ST4.03ch03	644,028	639,862	430	48,782.81	8.28
*StGLP58*	PGSC0003DMT400067755	40,482,716	40,483,336	ST4.03ch11	40,483,336	40,482,716	206	21,854.14	5.51
*StGLP59*	PGSC0003DMT400061586	5,333,291	5,333,929	ST4.03ch11	5,333,929	5,333,291	212	23,300.94	6.41
*StGLP60*	PGSC0003DMT400067758	40,480,217	40,480,828	ST4.03ch11	40,480,828	40,480,217	203	21,650.87	6.91
*StGLP61*	PGSC0003DMT400067709	40,477,347	40,479,544	ST4.03ch11	40,479,544	40,477,347	303	32,003.43	6.34
*StGLP62*	PGSC0003DMT400003235	5,633,348	5,633,974	ST4.03ch11	5,633,974	5,633,348	208	22,581.1	9.36
*StGLP63*	PGSC0003DMT400040214	45,351,028	45,352,678	ST4.03ch11	45,352,678	45,351,028	356	38,205.97	6.15
*StGLP64*	PGSC0003DMT400070424	43,975,664	43,979,229	ST4.03ch11	43,979,229	43,975,664	509	57,013.7	5.67
*StGLP65*	PGSC0003DMT400040162	45,354,585	45,358,874	ST4.03ch11	45,358,874	45,354,585	513	56,512.04	6.58
*StGLP66*	PGSC0003DMT400092000	40,481,535	40,482,146	ST4.03ch11	40,482,146	40,481,535	203	21,422.5	6.26
*StGLP67*	PGSC0003DMT400029687	58,884,139	58,885,066	ST4.03ch10	58,885,066	58,884,139	217	22,866.41	7.72
*StGLP68*	PGSC0003DMT400092672	58,892,246	58,892,689	ST4.03ch10	58,892,689	58,892,246	147	16,028.43	6.16
*StGLP69*	PGSC0003DMT400029689	58,881,482	58,882,474	ST4.03ch10	58,882,474	58,881,482	217	23,082.58	7
*StGLP70*	PGSC0003DMT400087849	58,876,226	58,879,838	ST4.03ch10	58,879,838	58,876,226	429	45,493.44	6.45

*CDS, coding sequence; pI, isoelectric point.*

### Chromosomal Locations, Synteny, and Gene Duplication Analysis

**FIGURE 3:**
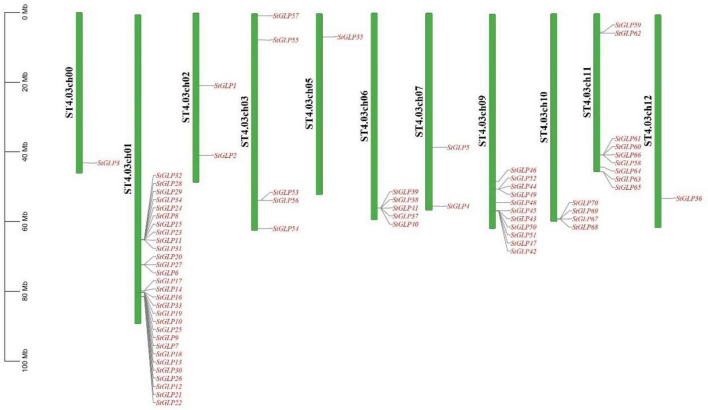
Chromosomal distribution of *StGLP* genes. Green color shows chromosomes, red color shows *StGLP* genes, and black color shows the number of chromosomes.

**FIGURE 4:**
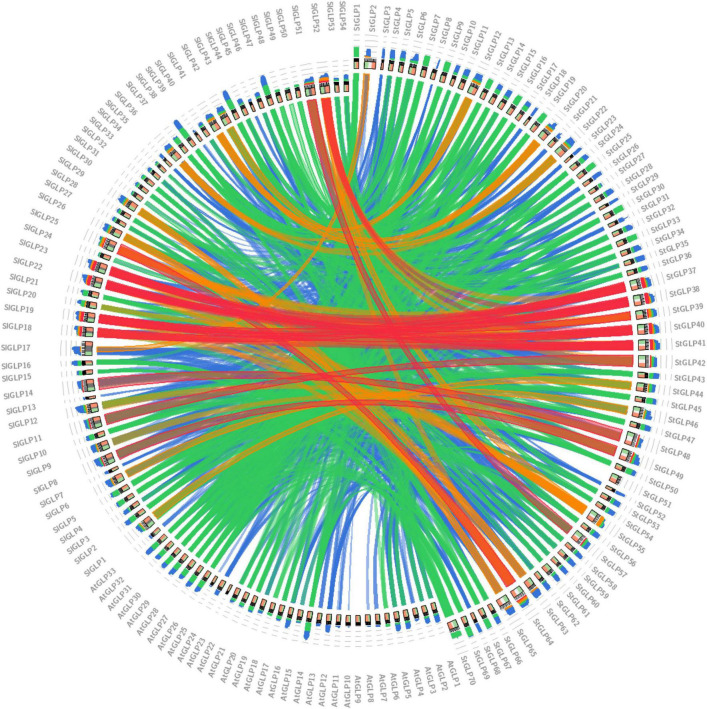
Synteny analysis of GLPs among potato, tomato, and *Arabidopsis* genomes. In comparative synteny analysis, the color and intensity of inward and outward tangling of ribbons showed conservation and duplication events, respectively.

Chromosomal distribution analysis of *StGLPs* depicted their unequal localization on all chromosomes (Figure 3). A maximum number of 29 genes were present on the 1st chromosome, while the 9th chromosome had 11 genes. There are 9 genes present on chromosome number 11. Only 2 genes are located on chromosome number 02 (Figure 3) in our results. Furthermore, there was no gene located on chromosome number 08. To determine the rate of molecular evolution of each gene pair that has been duplicated, the Ka to Ks (synonymous/non-synonymous substitution) ratios were calculated. When the value of the ratio is more than 1, then the selection effect will be positive; if its value is less than 1, then purifying selection will be exhibited, while when this ratio’s value is equal to 1, then there will be the presence of neutral selection in gene pairs (Yang and Bielawski, 2000). The cosmic majority of *StGLPs* genes exhibited a value of Ks more than 0.52, and the corresponding deviation time may be more than 100 million years ago (MYA). In this study, duplicated genes (*StGLP4/StGLP35*) showed the Ks value 3.54 while its corresponding time may be 682.30 MYA (Table 2). Comparative synteny analysis was analyzed among *A. thaliana*, *S. tuberosum*, and *S. lycoperiscum* which exhibited a phenomenal relationship in the evolution expression, duplication, triplication, and function of genes. Genes sequence of *SlGLP17* exhibited synteny with the sequence of *StGLP2*. At the same time, two potato genes, *StGLP20* and *StGLP22*, showed synteny with the *SlGLP41* gene of tomato. The comparative synteny analysis revealed the conservation and duplication of inward and outward tangling events in ribbons, respectively (Figure 4).

**TABLE 2:** Gene duplication and selection pressure of *StGLPs*.

Seq_1	Seq_2	Ka	Ks	Ka_Ks	Time (MYA)
*StGLP6*	*StGLP20*	0.00587159	0.019568986	0.300045672	3.763266576
*StGLP12*	*StGLP22*	0.059038628	0.193459314	0.305173356	37.20371428
*StGLP63*	*StGLP65*	0.047638432	0.282922672	0.168379692	54.40820612
*StGLP42*	*StGLP57*	0.321196935	1.332395831	0.241067202	256.2299675
*StGLP39*	*StGLP55*	0.237145767	0.571605039	0.41487697	109.924046
*StGLP38*	*StGLP41*	0.06693679	0.288679494	0.231872342	55.51528724
*StGLP4*	*StGLP35*	0.635162456	3.548006451	0.179019533	682.3089328
*StGLP36*	*StGLP54*	0.090222715	1.503069078	0.060025661	289.0517458
*StGLP3*	*StGLP62*	0.083490733	0.188993779	0.441764451	36.34495755
*StGLP58*	*StGLP61*	0.022084776	0.136249525	0.162090661	26.20183183
*StGLP60*	*StGLP66*	0.066557407	0.157685536	0.422089489	30.32414157
*StGLP14*	*StGLP17*	0.012705439	0.132943691	0.095570077	25.56609445
*StGLP53*	*StGLP56*	0.063376386	0.255022102	0.248513307	49.04271201
*StGLP67*	*StGLP70*	0.023344121	0.08530024	0.273670051	16.40389238
*StGLP19*	*StGLP33*	0.021457901	0.147797879	0.14518409	28.42266902
*StGLP1*	*StGLP30*	0.019660366	0.228158531	0.086169759	43.87664054
*StGLP18*	*StGLP26*	0.267626689	0.427706455	0.625725158	82.25124131
*StGLP7*	*StGLP13*	0.002038737	0.033904078	0.060132509	6.520014918
*StGLP43*	*StGLP50*	0.124561464	0.128964836	0.965856028	24.80093
*StGLP11*	*StGLP28*	0.149905888	0.166366256	0.901059457	31.99351068
*StGLP31*	*StGLP32*	0.119090894	0.142681101	0.834664809	27.43867323
*StGLP29*	*StGLP34*	0.206352764	0.292304449	0.7059515	56.21239411

*Ka, number of non-synonymous substitutions per non-synonymous site; Ks, number of synonymous substitutions per synonymous site; MYA, million years ago.*

### *Cis*-Elements and *miRNA* Analysis

**FIGURE 5:**
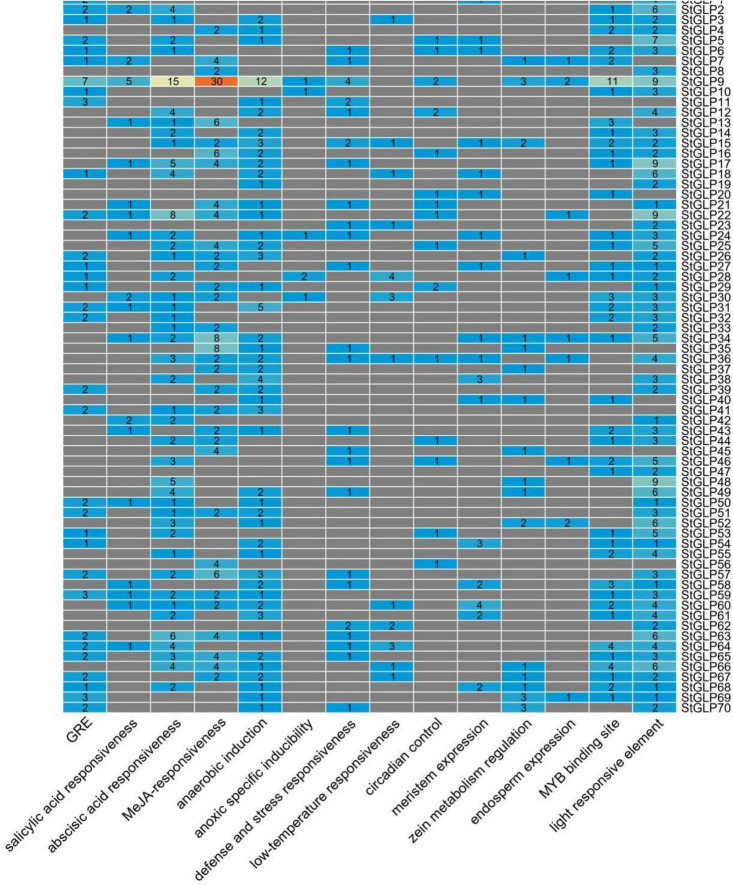
*Cis*-acting elements distribution in the upstream regulatory regions of *StGLPs*.

To observe the *StGLPs* gene function and their regulatory role, promoter *cis*-elements were analyzed using the PlantCARE database. The *cis*-elements that are associated with plant hormones were identified. As shown in Figure 5, the number of genes associated with phytohormones indicates the importance of these genes in phytohormone stress. Furthermore, the role of anaerobic, light, and low-temperature responsive factors in this study was discovered (Figure 5). Mainly several elements that are responsive to light were categorized to be extensively distributed in all genes, indicating the significant role of *StGLPs* in response to light stress. Generally, findings envisaged that diversification in the expression levels of various *StGLPs* might be attributed to different plant hormones and environmental stress. The Hormones- and stress-related cis-elements found in the promoter regions of StGLPs are given in Supplementary Table 4.

**FIGURE 6:**
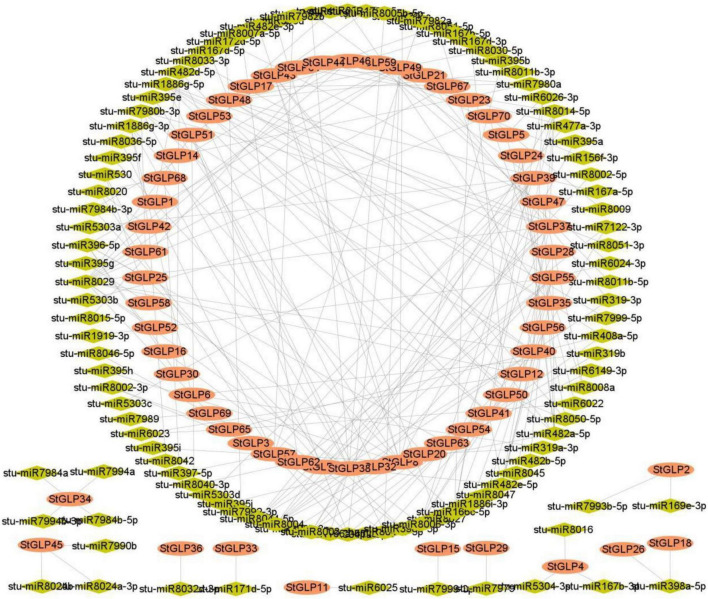
miRNAs targeting *StGLP* genes. Network figure of anticipated miRNAs targeting the *StGLP* genes. Green color represents miRNAs, while orange color corresponds to the target *StGLP* gene.

Many researchers have reported that microRNA mediated a significant role in enhancing stress response in plants (Su et al., 2021; Xu et al., 2021). Thus, for an in-depth comprehension of miRNA-triggered post-transcriptional modulations of *StGLPs*, we identified miRNAs targeting *StGLPs* genes. Several sites that are targeted by miRNA are illustrated in Supplementary Table 1. Our findings revealed that seven members of the miR166 family target six genes, including *StGLP8, StGLP54, StGLP23, StGLP24, StGLP38*, and *StGLP28*. A gene, *StGLP2*, was targeted by a single member of the miR169 family. Targets *StGLP51, StGLP48*, and *StGLP47* (Figure 6 and Supplementary Table 5) are all three members of the miRNA gene family. The findings of our study revealed that two members of the miR8040 aimed at two genes, *StGLP51* and *StGLP62*. A gene *StGLP55* was the target of two members of the miR1886 family. Our results mainly estimated two genes, *StGLP40* and *StGLP8*, to target numerous miRNAs.

**FIGURE 7:**
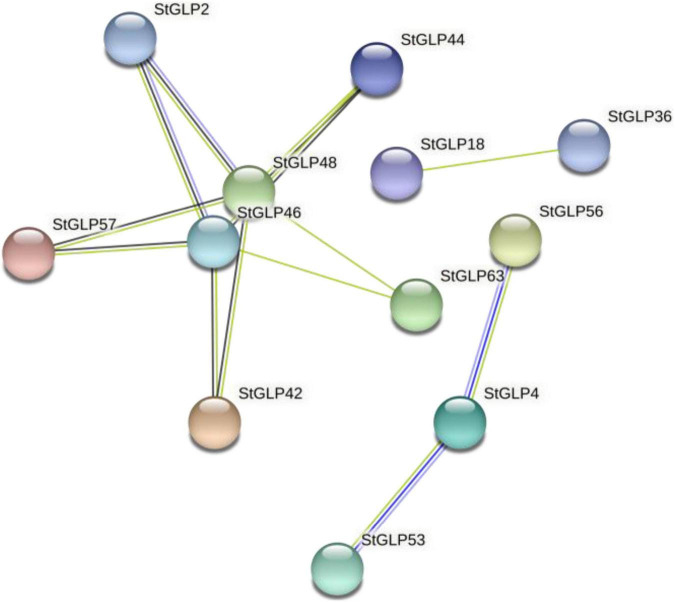
Protein–protein interaction network of *StGLPs*.

The protein–protein interaction network provided details about StGLPs in the genome of potatoes. The interaction network was observed among several proteins, including StGLP57, StGLP44, StGLP46, StGLP48, and StGLP2. These proteins depicted the concurrence, co-expression, fusion, and homology of genes. Likewise, another framework was also identified among StGLP53, StGLP63, and StGLP4 protein. Among all StGLPs, two proteins, StGLP48 and StGLP46, are responsible for framing a protein–protein interaction network, so these can be reputed as hub proteins of StGLPs (Figure 7).

### Tissue-Specific Expression Profiling

**FIGURE 8:**
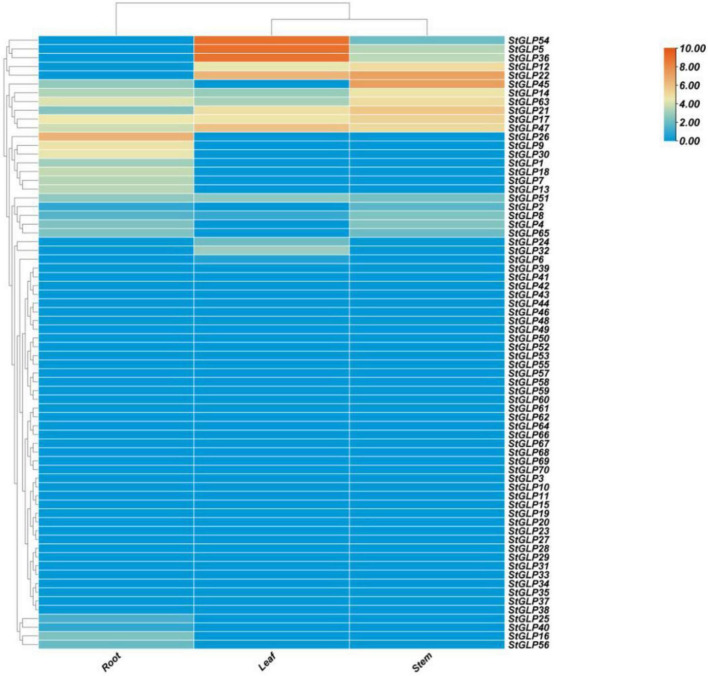
Expression profiling of *StGLP* genes in leaves, roots, and stems. The orange and blue colors display high- to low-expression levels. The expression heat map was created by taking log10 normalized values of fragments per kilobase million (FPKM).

Expression profiling of *StGLPs* revealed that many genes exhibited high expression in the tissues of roots than that in the tissues of stems and leaves. Some genes showed expression in some tissues, while some genes have no expression in others. For example, the *StGLP17* gene showed a higher expression level in the tissues of roots, leaves, and stems, while the *StGLP26* and *StGLP45* genes showed higher expression levels in the tissues of roots and stems. Furthermore, in the tissues of leaves, *StGLP5* genes showed the maximum expression in our results (Figure 8).

### Expression Patterns of StGLP Genes in Response to Heat and Salt Stress

**FIGURE 9:**
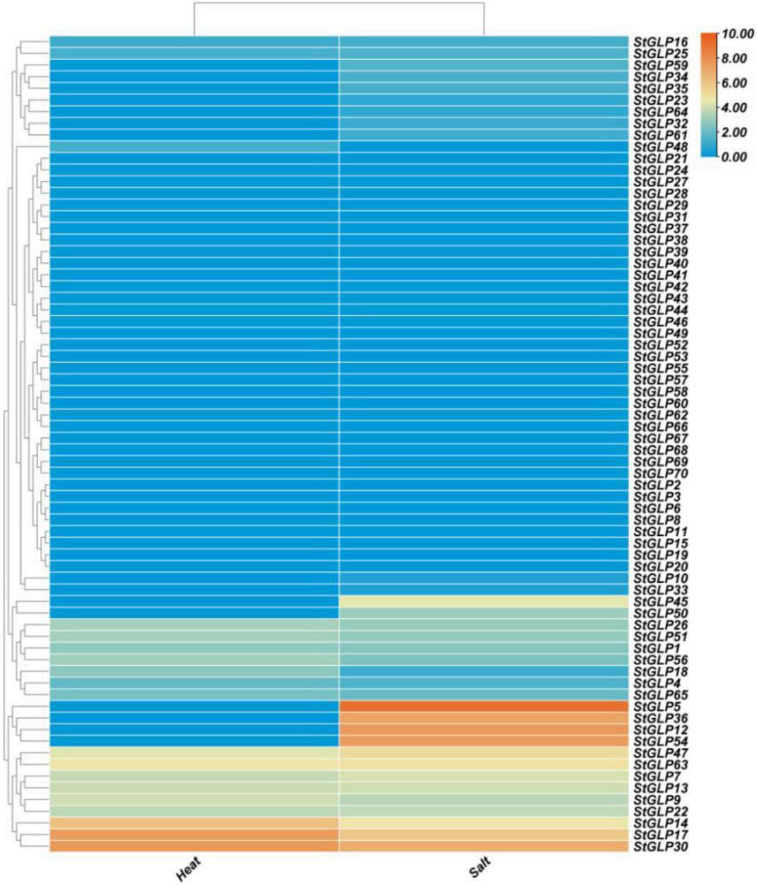
Expression profiling of *StGLP* genes in response to salt and heat treatments. The orange and blue colors display high- to low-expression levels. The expression heat map was created by taking log10 of fragments per kilobase million (FPKM).

Transcriptional profiling of *GLPs* was performed when potato plants were subjected to heat stress. *StGLPs* may help potato plants cope better with high temperatures, according to some research. A total of 19 *StGLPs* genes were expressed in response to heat stress. Moreover, three genes, *StGLP30*, *StGLP17*, and *StGLP14*, exhibited a relatively higher expression level in potatoes after heat treatment (Figure 9). In our results, *StGLP30* gene expression was higher than *StGLP17* and *StGLP14*.

Furthermore, *StGLP16* genes showed the lowest expression compared with other expressed *StGLPs* after heat treatment. Likewise, 29 *GLPs* were expressed in response to salt treatment. This study showed that *StGLP54*, *StGLP12*, and *StGLP5* genes were highly expressed in potatoes after salt treatment (Figure 9), but the expression level of the *StGLP5* gene was relatively higher as compared to *StGLP12* and *StGLP54* genes in the potato genome.

### Expression Patterns of StGLP Genes in Response to Phytohormones

Three hormones, abscisic acid (ABA), gibberellic acid (GA3), and indole acetic acid (IAA), were selected to evaluate the hormonal response of StGLPs.

**FIGURE 10:**
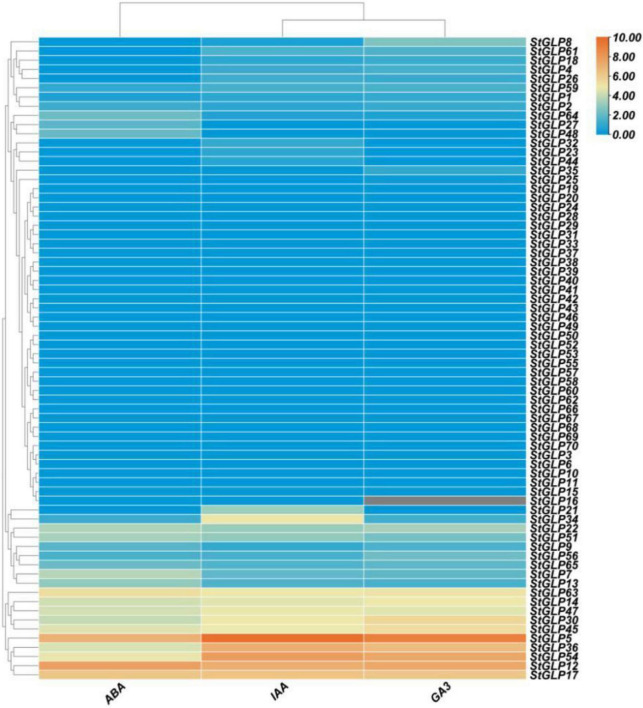
Expression profiling of *StGLP* genes in response to ABA, IAA, and GA3. The orange and blue colors display high- to low-expression levels. The expression heat map was created by taking log10 of fragments per kilobase million (FPKM).

This study results showed that 22 *StGLP* genes were expressed after the treatment of ABA. Similarly, in the case of IAA treatment, 24 *StGLP* genes were expressed. Furthermore, the *StGLP12* gene was highly expressed compared with others after the treatment of ABA, while *StGLP5* showed the highest expression after IAA treatment (Figure 10). Additionally, 27 *StGLPs* were expressed when treated with GA3, and the expression of gene *StGLP14* was highest among all 27 expressed genes (Figure 10). The upregulation of *StGLPs* after ABA, IAA, and GA3 treatment revealed the significant role of *StGLP* genes in mitigating the lethal effects of different hormonal and environmental stress.

### RT-qPCR Analysis

**FIGURE 11:**
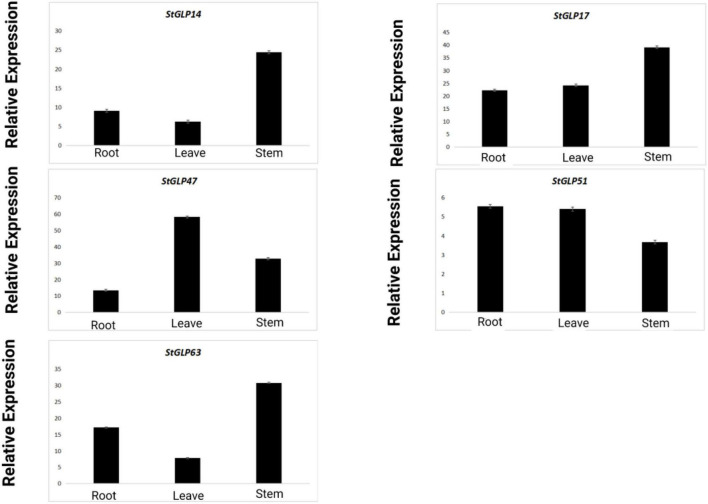
Real-time relative expressions of *StGLPs* in leaves, roots, and stems.

**FIGURE 12:**
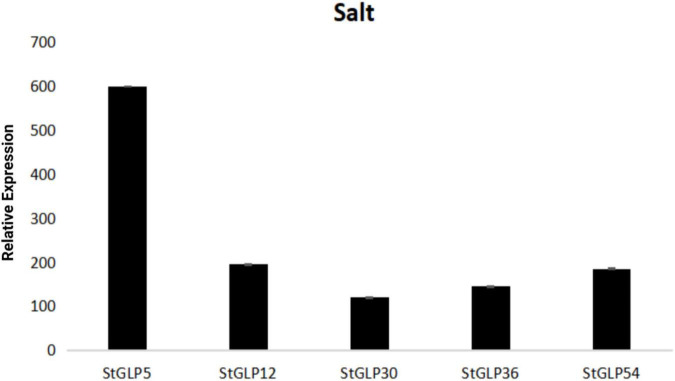
Real-time relative expressions of *StGLPs* in salt treatment.

Real-time-qPCR was performed to confirm the expression of five genes, namely, *StGLP63*, *StGLP17*, *StGLP51*, *StGLP14*, and *StGLP47*, in the tissues of roots, stems, and leaves (Figure 11). Two genes, namely, *StGLP17* and *StGLP14*, exhibited a comparatively higher expression level in the tissues of stems than that in the tissues of roots and leaves. In contrast, the expression of *StGLP47* was relatively more in the tissues of leaves than that in the tissues of roots and stems. Generally, findings depicted that *StGLP* genes may perform particular functions in potato plant growth and developmental process. Similarly, RT-qPCR was performed to confirm the expression of five genes, namely, *StGLP5*, *StGLP12*, *StGLP30*, *StGLP 36*, and *StGLP54*, in leaves treated with 150 mM NaCl. The results showed that the *StGLP5* genes showed the maximum expression after 24 h of NaCl stress. In addition, genes such as *StGLP12*, *StGLP 36*, and *StGLP54* were highly expressed with the extension of stress time. Moreover, *StGLP30* showed expression but low expression compared with the other four genes (Figure 12).

## Discussion

Potato is an integral part of the food chain worldwide (Zaynab et al., 2017b). Potato plants are subjected to different biotic and abiotic stresses (Zaynab et al., 2021b). *GLPs* have a significant role in plant signaling and response to various environmental stresses (Yuan et al., 2021). Members of *GLPs* show variations in their expression and possess considerable functions in several tissues at different phases of growth cycle of the plant (Barman and Banerjee, 2015). This crucial gene family carried out vital defense mechanisms and is an essential part of plant defense processes. The expression of the essential genes increases exponentially when plants encounter stress like salinity (Wang et al., 2013; Zaynab et al., 2021d), water shortage (Komatsu et al., 2010), heavy metals (Berna and Bernier, 1999), and heat stress (Qasim et al., 2018b). Various functions of *GLPs* have also been determined in different crops such as *Hordeum vulgare* (Zimmermann et al., 2006), *A. thaliana* (Rietz et al., 2012), *Oryza sativa* (Li et al., 2016), and *Glycine max* (Wang et al., 2014). But till now, there has been no considerable in-depth study of *GLPs* in the potato. These days, the whole-genomic sequence of potato is comfortably accessible, allowing us to conduct genome-wide analysis of the *GLP* genes family that may prove worthy for further research.

Structural analysis of *StGLPs* will prove beneficial for their functional characterization. The utilization of the evolutionary relic of *GLPs* has established that the evolution of genes has been affected by the arrangement of their exons and introns (Moore and Purugganan, 2005; Flagel and Wendel, 2009). This is in correlation with previous scientists finding that genes with short introns or no introns are retained in the genomic systems of plants during the evolutionary process. At the same time, the expression levels of such genes are also low in plants (Mattick and Gagen, 2001). Moreover, the compact arrangement of genes favors the expression response of the *GLPs* against different exo or endogenous stimuli (Jeffares et al., 2008). Our findings through structural analysis of these genes inferred that those sequences of *StGLP* depicted a similar number of exons–introns with identical functional properties because they may have arisen through duplication events in the course of evolution (Waqas et al., 2019).

Studying the defense mechanisms employed by plants against various types of environmental stress, *cis*-elements were investigated in the promoter region of these genes. According to our findings, different *cis*-elements were identified that show their response against light and hormone stress. Several identified *cis*-elements were associated with ABA, SA, GA, MeJA, low temperature, anaerobic induction, and light. Earlier reports have attributed the stress response of plants to these *cis*-acting elements (Osakabe et al., 2014). *Cis*-factors are integral parts of plant stress response machinery as they regulate stress-responsive genes (Wu et al., 2014). So, these crucial *Cis*-acting sites of *StGLPs* indicate their behavior when different stresses are imposed.

All identified *StGLP* genes were classified into six clades. Similarly, phylogenetic grouping was also observed in other plants. The comparative phylogenetic analysis revealed that the organization of *S. lycoperiscum*, *S. tuberosum*, and *A. thaliana* proteins was relatively similar in six clades, indicating that all *StGLP* genes in these groups may have descended from a common ancestor. Previous studies (Li et al., 2016) reported the classification of *GLPs* into six clades in rice and *Arabidopsis*, supporting our results.

The size of the genome, duplication events in the genome, and the distribution of genes are major determinants of genetic variability in land plants. Genetic duplication property has long been recognized throughout the origins of evolutionary novelty, expression, and complexity of the gene families. We also noticed some duplications in *StGLPs* that may carry out vital roles in their amplification. As gene duplication is an imperative character in neofunctionalization, expansion, and variability among gene families (Lavin et al., 2005), similarly, the arrangement and localization of *StGLP* genes at chromosomes will enable breeders of potato to breed better varieties with desirable features.

Several types of research have depicted the response of plants to salinity and other abiotic stresses, and it is now well understood that miRNAs and their objects have direct impacts on the stress tolerance processes of plants (Li, 2015; Villanueva et al., 2016). This study recognized micro RNAs from various families targeting *StGLP* genes. Likewise, the unique role of miR156 has been widely documented during various abiotic stress in several plants (Cui et al., 2014; Kohli et al., 2014). Similarly, a study on cotton established a critical role of miR827 in response to saline stress (Covarrubias and Reyes, 2010); miR167 has also been recognized as one of the significant role players in response to various stressed environmental conditions (Khraiwesh et al., 2012). Concisely, these results are in concordance with our findings that revealed that miR156, miR167, and miR827 are significant determinants of tolerance in plants against several stresses as they alter the expression of *StGLP* genes in the genome of potatoes.

Germin-like gene miRNAs are generally present in embryos, cotyledons, leaves, stems, flowers, roots, and seeds. Some are expressed in response to different environmental conditions; this depends upon genes under analysis or species in which they are expressed. Many authentications have indicated that various *GLPs* may have a critical role in the defense mechanisms of plants (Breen and Bellgard, 2010). So, the expression profiling of *GLPs* in the potato is helpful for their in-depth comprehension. Many genes exhibited a high level of expression in tissues, suggesting the putative role of these genes in potato growth and developmental processes. Our RT-qPCR findings revealed that the upregulation of genes in the tissues of roots and stems indicated the role of *GLP* in potato plant growth.

Furthermore, *GLPs* expression was observed in response to hormones and abiotic stress. These findings are following earlier reports that GLPs are an integral part of the defense system in saline tolerance (Wang et al., 2013), drought stress (Komatsu et al., 2010), cold tolerance, and heavy metals tolerance (Berna and Bernier, 1999). Up till now, numerous researches have depicted that *GLPs* are significant players of plant stress response mechanisms. In our results, *StGLP14, StGLP17*, and *StGLP30* are upregulated in response to heat stress, and *StGLP5*, *StGLP12*, and *StGLP54* are highly expressed in response to salt stress supported by the findings of Wang et al. (2013). Hormones may alter the physio-biochemical pathways or metabolisms of plants by different pathways of signal transduction (Zaynab et al., 2017a; Fatima et al., 2021). ABA and IAA are major hormones of the immune system in plants. Several studies have established that besides being part of plant response to different stresses, GLPs also are involved in developing systems and hormonal signaling (Wang et al., 2020). To study how hormone signaling affects the production of StGLPs, leaves of potato were treated with ABA, GA3, and IAA, and the expression of genes was investigated. There was the induction of 22 genes on the treatment of ABA, 24 genes on the treatment of IAA, and 27 genes on the treatment of GA3, suggesting that various members of *StGLPs* carry out crucial roles in the plant defense system induced by these hormones. When the treatment of ABA and IAA was made, increasing the expression level of potato GLPs exhibited that these hormones possess an essential part in this defense mechanism, similar to the results obtained by Wang et al. (2020). The strong correlation between clusters of genes and their expression was noted under various stresses and tissues.

## Conclusion

In this study, we identified 70 *GLPs* in the genome of the potato by genome-wide analysis. The gene structure, synteny, phylogenetic motifs, promotor, and miRNA analysis were performed to enhance our comprehension. The expression profiling after various stresses has been analyzed. The findings exhibited that *GLPs* genes responded significantly against hormonal and abiotic stresses, strengthening our comprehension. In our results, the *StGLP5* gene showed the highest expression in response to salt stress. So, further analysis is needed to corroborate the persistent role of *GLPs* genes in response to salt stress.

## Data Availability Statement

The original contributions presented in the study are included in the article/Supplementary Material, further inquiries can be directed to the corresponding author.

## Author Contributions

MZ, JP, and YS gave the idea. MF, MA, and RA-Y performed the experiments. KK and SA wrote the manuscript. SL revised the manuscript. All authors contributed to the article and approved the submitted version.

## Conflict of Interest

The authors declare that the research was conducted in the absence of any commercial or financial relationships that could be construed as a potential conflict of interest.

## Publisher’s Note

All claims expressed in this article are solely those of the authors and do not necessarily represent those of their affiliated organizations, or those of the publisher, the editors and the reviewers. Any product that may be evaluated in this article, or claim that may be made by its manufacturer, is not guaranteed or endorsed by the publisher.

## Footnotes

1
https://phytozome.jgi.doe.gov/pz/portal.html

2
http://www.arabidopsis.org/

3
http://pfam.xfam.org/

4
http://www.hmmer.org/

5
http://web.expasy.org/protparam/

6
https://megasoftware.net/home

7
https://github.com/CJ-Chen/TBtools

8
https://meme-suite.org/meme/db/motifs

9
tools.bat.infspire.org/circoletto/

10
http://bioinformatics.psb.ugent.be/webtools/plantcare/html/

11
https://www.zhaolab.org/psRNATarget/

12
https://string-db.org/
