# Corrigendum: Genome-Wide Identification and Expression Profiling of Germin-Like Proteins Reveal Their Role in Regulating Abiotic Stress Response in Potato

**DOI:** 10.3389/fpls.2022.910487

**Published:** 2022-05-23

**Authors:** Madiha Zaynab, Jiaofeng Peng, Yasir Sharif, Mahpara Fatima, Mohammed Albaqami, Rashid Al-Yahyai, Khalid Ali Khan, Saqer S. Alotaibi, Ibrahim A. Alaraidh, Hassan O. Shaikhaldein, Shuangfei Li

**Affiliations:** ^1^Shenzhen Key Laboratory of Marine Bioresource and Eco-Environmental Sciences, College of Life Sciences and Oceanography, Shenzhen University, Shenzhen, China; ^2^Instrument Analysis Center, Shenzhen University, Shenzhen, China; ^3^College of Plant Protection, Fujian Agriculture and Forestry University, Fuzhou, China; ^4^College of Agriculture, Fujian Agriculture and Forestry University, Fuzhou, China; ^5^Department of Biology, Faculty of Applied Science, Umm Al-Qura University, Makkah, Saudi Arabia; ^6^Department of Plant Sciences, College of Agricultural and Marine Sciences, Sultan Qaboos University, Muscat, Oman; ^7^Research Center for Advanced Materials Science (RCAMS), King Khalid University, Abha, Saudi Arabia; ^8^Unit of Bee Research and Honey Production, Faculty of Science, King Khalid University, Abha, Saudi Arabia; ^9^Department, Faculty of Science, King Khalid University, Abha, Saudi Arabia; ^10^Department of Biotechnology, College of Science, Taif University, Taif, Saudi Arabia; ^11^Botany and Microbiology Department, Science College, King Saud University, Riyadh, Saudi Arabia

**Keywords:** abiotic stress, gene expression, gene structure, miRNA, RNA-seq, stress responses

Ali Raza was included as an author in the submitted article. However, during proofing the authors submitted the authorship change form but the typesetter missed the author name removal request. The corrected Author Contributions Statement and Funding appears below.

Madiha Zaynab^1†^, Jiaofeng Peng^2†^, Yasir Sharif^3^, Mahpara Fatima^4^, Mohammed Albaqami^5^, Rashid Al-Yahyai^6^, Khalid Ali Khan^7, 8, 9^, Saqer S. Alotaibi^10^, Ibrahim A. Alaraidh^11^, Hassan O. Shaikhaldein^11^ and Shuangfei Li^1*^

^1^ Shenzhen Key Laboratory of Marine Bioresource and Eco-Environmental Sciences, College of Life Sciences and Oceanography, Shenzhen University, Shenzhen, China

^2^ Instrument Analysis Center, Shenzhen University, Shenzhen, China

^3^ College of Plant Protection, Fujian Agriculture and Forestry University, Fuzhou, China

^4^ College of Agriculture, Fujian Agriculture and Forestry University, Fuzhou, China

^5^ Department of Biology, Faculty of Applied Science, Umm Al-Qura University, Makkah, Saudi Arabia

^6^ Department of Plant Sciences, College of Agricultural and Marine Sciences, Sultan Qaboos University, Muscat, Oman

^7^ Research Center for Advanced Materials Science (RCAMS), King Khalid University, Abha, Saudi Arabia

^8^ Unit of Bee Research and Honey Production, Faculty of Science, King Khalid University, Abha, Saudi Arabia

^9^ Department, Faculty of Science, King Khalid University, Abha, Saudi Arabia

^10^ Department of Biotechnology, College of Science, Taif University, Taif, Saudi Arabia

^11^ Botany and Microbiology Department, Science College, King Saud University, Riyadh, Saudi Arabia

## Author Contributions

MZ, JP, and YS gave the idea. MF, MA, and RA-Y performed the experiments. KK and SA wrote the manuscript. SL revised the manuscript. All authors contributed to the article and approved the submitted version.

## Funding

This work was supported by Shenzhen Special Project for Sustainable Development (KCXFZ20201221173211033). The authors appreciate the support of the Research Center for Advanced Materials Science (RCAMS) at King Khalid University Abha, Saudi Arabia, through a grant KKU/RCAMS/G002-21. Taif University Researchers Supporting Project number (TURSP-2020/38), Taif University, Taif, Saudi Arabia.

The authors and production team apologize for this error and state that this does not change the scientific conclusions of the article in any way. The original article has been updated.

## Publisher's Note

All claims expressed in this article are solely those of the authors and do not necessarily represent those of their affiliated organizations, or those of the publisher, the editors and the reviewers. Any product that may be evaluated in this article, or claim that may be made by its manufacturer, is not guaranteed or endorsed by the publisher.

