# Supplementary material for: Genome-Wide Identification and Expression Profiling of Germin-Like Proteins Reveal Their Role in Regulating Abiotic Stress Response in Potato
Source: Front Plant Sci. 2022 Feb 17;12:831140. doi: 10.3389/fpls.2021.831140 (PMC8891383; doi:10.3389/fpls.2021.831140)
Supplement: Supplementary file 1 [file Table_1.docx]

Supplementary Table 1: Primers used in the study.

| Gene | Primer | Sequence |
| --- | --- | --- |
| *EF-1α* | F | GGCTTGATGACACCAGTT |
|  | R | ACCATACCAGCATCACCGTTCT |
| Primers to check the tissue specific expression | | |
| *StGLP14* | F | TCCAGCAGAACAATGGGGATG |
|  | R | TGGTGATGAAGCGAACAACG |
| *StGLP17* | F | ATGGCTGTGGTGGCTATGTT |
|  | R | CTTTGCTATGGCCATTGATG |
| *StGLP47* | F | TGCAAAGACGCAAAGCTAGC |
|  | R | TGTTGGTGTTGGCATTCCAC |
| *StGLP51* | F | ACTCCTTGGGTTTGCCAATC |
|  | R | AGCACACGAAACGAAGAAGG |
| *StGLP63* | F | ACCAAAACTGCACACAAGGC |
|  | R | ACCAGTGAAAATCCCGTTGG |
| Primers to check the salt stress induced expression | | |
| *StGLP5* | F | ACGCTTGCAAGAAACCATCC |
|  | R | TTGAGGAGCAAAAGCAGGAG |
| *StGLP12* | F | TCCGTTTGTTGTGGAAGCTG |
|  | R | AGATGCTGCTTCCCAAACTG |
| *StGLP30* | F | TTTCAAACCCGGGTCCAAAC |
|  | R | AGTCCAGCAAATGCAACAGC |
| *StGLP36* | F | AGCAGACTTGAAAGGACCAGAG |
|  | R | TGCAAATGCTGGTGACACTG |
| *StGLP54* | F | ATTTGCGGCCCAATTTCCAG |
|  | R | ACCTGCGGCCAAATCTAAAC |
